# Genome-wide profiling of long noncoding RNAs involved in wheat spike development

**DOI:** 10.1186/s12864-021-07851-4

**Published:** 2021-07-02

**Authors:** Pei Cao, Wenjuan Fan, Pengjia Li, Yuxin Hu

**Affiliations:** 1 Key Laboratory of Plant Molecular Physiology, CAS Center for Excellence in Molecular Plant Sciences, Institute of Botany, Chinese Academy of Sciences, 100093 Beijing, China; 2 University of Chinese Academy of Sciences, 100049 Beijing, China; 3 National Center for Plant Gene Research, 100093 Beijing, China

**Keywords:** lncRNAs, miRNAs, Spike, Transcriptome, Wheat

## Abstract

**Background:**

Long noncoding RNAs (lncRNAs) have been shown to play important roles in the regulation of plant growth and development. Recent transcriptomic analyses have revealed the gene expression profiling in wheat spike development, however, the possible regulatory roles of lncRNAs in wheat spike morphogenesis remain largely unclear.

**Results:**

Here, we analyzed the genome-wide profiling of lncRNAs during wheat spike development at six stages, and identified a total of 8,889 expressed lncRNAs, among which 2,753 were differentially expressed lncRNAs (DE lncRNAs) at various developmental stages. Three hundred fifteen differentially expressed *cis-* and *trans-*regulatory lncRNA-mRNA pairs comprised of 205 lncRNAs and 279 genes were predicted, which were found to be mainly involved in the stress responses, transcriptional and enzymatic regulations. Moreover, the 145 DE lncRNAs were predicted as putative precursors or target mimics of miRNAs. Finally, we identified the important lncRNAs that participate in spike development by potentially targeting stress response genes, TF genes or miRNAs.

**Conclusions:**

This study outlines an overall view of lncRNAs and their possible regulatory networks during wheat spike development, which also provides an alternative resource for genetic manipulation of wheat spike architecture and thus yield.

**Supplementary Information:**

The online version contains supplementary material available at 10.1186/s12864-021-07851-4.

## Background

The architecture of flowering plants is largely influenced by the varied inflorescences and their spatial positions and orientations [1]. The inflorescence development begins with the transition of a vegetative shoot apical meristem (SAM) to a reproductive inflorescence meristem (IM) after perceiving intrinsic and extrinsic signals, such as photoperiod and temperature [2, 3]. In the model eudicot plant *Arabidopsis*, the IM directly generates floral meristem (FM), which subsequently produces floral organ primordia and eventually develops into a simple raceme-type inflorescence [4]. By contrast, in monocot crop such as wheat, the IM initiates spikelet meristem (SM) and FM, and finally forms a raceme-like unbranched inflorescence named spike. Although accumulating evidence has demonstrated that some key regulators governing inflorescence development, including the well-known ABCDE model genes for floral organ development, are functionally conserved among different plant species [5, 6], the largely diversified morphology of inflorescences in plant kingdom suggests that some divergent regulators or mechanisms may exist and contribute to specific inflorescence architecture among different plant species and crops.

As the reproductive organ, wheat spike is tightly related to agronomic traits and thus yield. For example, the grain number per spike is one of the crucial determinants of the yield [7]. The wheat spike development involves the subsequent formation and development of SM, glume primordium, FM, stamen and pistil primordia, and floral organs [8]. Recently, several important regulators governing wheat spike development have been characterized. For examples, the *TaTFL1-2D*, an ortholog of *Arabidopsis TERMINAL FLOWER 1* (*TFL1*), regulates wheat spike complexity by altering the number of spikelet, floret, and grain per spike [9], while the *VERNALIZATION 1* (*VRN1*, also named as *FRUITFULL 1* (*FUL1*), *FUL2* and *FUL3* function redundantly in determining SM identity, as the lateral meristems in the *vrn1 ful2 ful3* triple mutant are reverted to vegetative meristems to form leaves instead of spikelets [10]. The *FRIZZY PANICLE (FZP)* modifies the spike structure by regulating SM identity, since its mutation results in the ectopic formation of SM and thus supernumerary spikelets [11]. Moreover, recent studies have begun to reveal the gene expression profiling during wheat spike development. The transcriptome of wheat inflorescence highlighted the gene regulatory networks between miRNAs and their target genes [12], and the gene expression profiles at six developmental stages of wheat spike revealed the expression dynamics of some important genes regulating spike development, including *VRN1*, *Photoperiod-1* (*Ppd-1*) and *Earliness per se 3* (*Eps-3*) [13]. An associative transcriptome analysis of 90 wheat varieties also identified a few of genes related to spike complexity [9].

LncRNAs are defined as the RNA transcripts more than 200 base pairs that lack protein-encoding potential, which are less abundant but exhibit more spatiotemporally specific expression patterns than the protein-coding genes [14]. Previous studies have shown that lncRNAs can function in a *cis* manner by affecting neighboring loci, and in *trans* by performing distal regulatory functions, as scaffolds for protein complexes, decoys, guides, and enhancers to regulate gene expression [15, 16]. Moreover, some of lncRNAs are reported to function as the precursors of miRNAs to influence miRNA biosynthesis or as mimic targets and thus interfering miRNA functions [17]. In *Arabidopsis* and rice, lncRNAs have been reported to play essential roles in the development of reproductive organs, including floral transition, meiosis progression, anther and pollen development [18–22]. For examples, the *Arabidopsis COOLAIR* and *COLDAIR* influence flowering time by regulating *FLOWERING LOCUS C* (*FLC*) expression at epigenetic and posttranscriptional levels [18], while the *asHSFB2a* affects female gametophyte development [23]. The rice *long-day-specific male-fertility-associated RNA* (*LDMAR*) regulates anther development and male sterility in a photoperiod-sensitive manner [22]. Recent works also suggest that lncRNAs participate in grain development and stress response in wheat [24, 25]. However, the regulatory roles of lncRNAs during wheat spike development remain largely elusive.

Here, we analyzed the dynamic transcriptome of developing wheat spike, and identified a total of 8,889 expressed lncRNAs with 2,753 DE lncRNAs at different developmental stages. We further predicted 315 DE lncRNA-mRNA regulatory pairs and analyzed the molecular events regulated by these DE lncRNA. Subsequently, 24 important lncRNAs and their potential regulatory genes were identified. We also predicted some lncRNAs may function as putative precursors or target mimics of miRNAs. Our data thus provide an overall view on dynamic expression and potential regulation of lncRNAs during wheat spike development, which are also alternative resources for further manipulation of wheat spike architecture and thus yield.

## Results

### The lncRNAs transcriptomic profiling of developing wheat spike

To monitor the dynamic profiling of lncRNAs during wheat spike development, the developing spikes of winter wheat Zhengmai366 were collected at six stages (S1-S6) referred as IM stage (S1), SM stage (S2), glume primordium stage (S3), FM stage (S4), stamen and pistil primordium stage (S5) and floral organ stage (S6), respectively, according to anatomic and morphological features described previously [8] (Supplementary Fig. S1). These developmental stages morphologically corresponded to the W2.0 (S1), W2.5 (S2), W3.0 (S3), W3.5 (S4), W5 (S5) and W6 (S6) on the respective Waddington scale as described previously [26].

A total of 18 RNA samples from six developmental stages with three biological replicates were subjected to RNA isolation and sequencing, respectively. Approximately, 15 Gb clean base pairs for each sample were aligned to the wheat genomic sequence (IWGSC Refseq v1.0, Ensemble Plants; Supplementary Table S1). Unique reads were used to quantify gene models in fragments per kilobase of exon model per million mapped reads (FPKM). With average FPKM ≥ 1 of three replicates in at least one stage, a total of 48,345 coding genes were expressed, among which 11,792 were differentially expressed during spike development (Supplementary Table S2; Supplementary Table S3), consistent with ~ 50,000 genes detected in previous study [12].

**Fig. 1:**
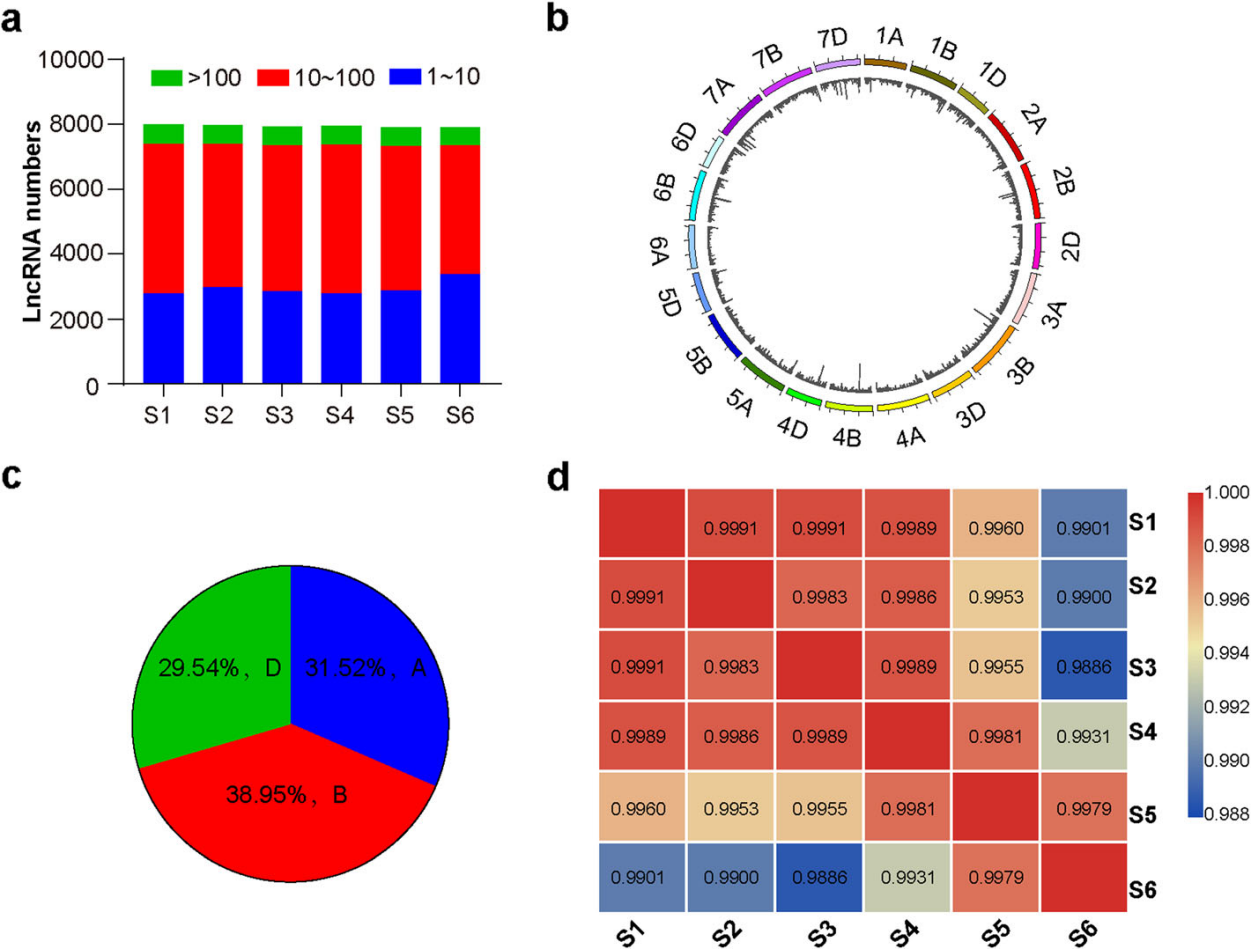
Overview of lncRNAs profiling in developing wheat spikes. **a** The numbers of expressed lncRNAs detected at each developmental stage of developing spikes. The S1-S6 represent the developmental stage of inflorescence meristem (IM), spikelet meristem (SM), glume primordium, floret meristem (FM), stamen and pistil primordia, and floral organ (anther and awn), respectively. LncRNAs with different expression abundance (TPM) are shown in different colors. **b** Chromosome distribution of expressed lncRNAs during wheat spike development. **c** Subgenome distribution of lncRNAs in wheat A, B and D genomes. **d** The correlation coefficients of detected lncRNAs among the six developmental stages from S1 to S6

Following LGC (https://bigd.big.ac.cn/lgc/), Coding-Non-Coding-Index (CNCI) predictions and Pfam blast of potential coding capacity, a total of 8,889 transcripts with average FPKM ≥ 1 in at least one stage were considered as the high confidence lncRNAs expressed during spike development, among which 143 had been annotated in the total of 362 lncRNAs annotated in wheat genome (Supplementary Table S4). For individual developmental stage, there were ~ 8,000 lncRNAs, including ~ 3,000 lncRNAs with low abundance (transcripts per kilobase of exon model per million mapped reads, TPM value between 1 ~ 10), ~ 4,500 lncRNAs with medium abundance (TPM value between 10 ~ 100), and ~ 500 lncRNAs with high abundance (TPM value > 100) (Fig. 1a). These expressed lncRNAs were almost evenly distributed across all chromosomes of the wheat genome (Fig. 1b). However, the subgenome distribution showed that lncRNAs appeared to be slightly preferential on B subgenome (A, 2,677, 31.52 %; B, 3,308, 38.95 %; D, 2,509, 29.54 %; Fig. 1c). Moreover, the lncRNAs expression levels between any two developmental stages were highly correlated with the correlation coefficient above 0.98 (Fig. 1d), consistent with the continuity of spike development despite of distinct morphological characteristics existed at each stage. Importantly, among the expressed lncRNAs, 7,522 (84.7 %) were detected in all the six developmental stages (Supplementary Fig. S2). The numbers of lncRNAs expressed at only one stage (FPKM < 1 at the other stages) were 63, 70, 60, 43, 41 and 61 from S1 to S6, respectively (Supplementary Fig. S2; Supplementary Table S5). These observations demonstrate that lncRNAs are highly involved in spike development and some of them may function in a stage-specific manner to contribute to specific developmental characteristic.

### Expression clustering of differentially expressed lncRNAs

**Fig. 2:**
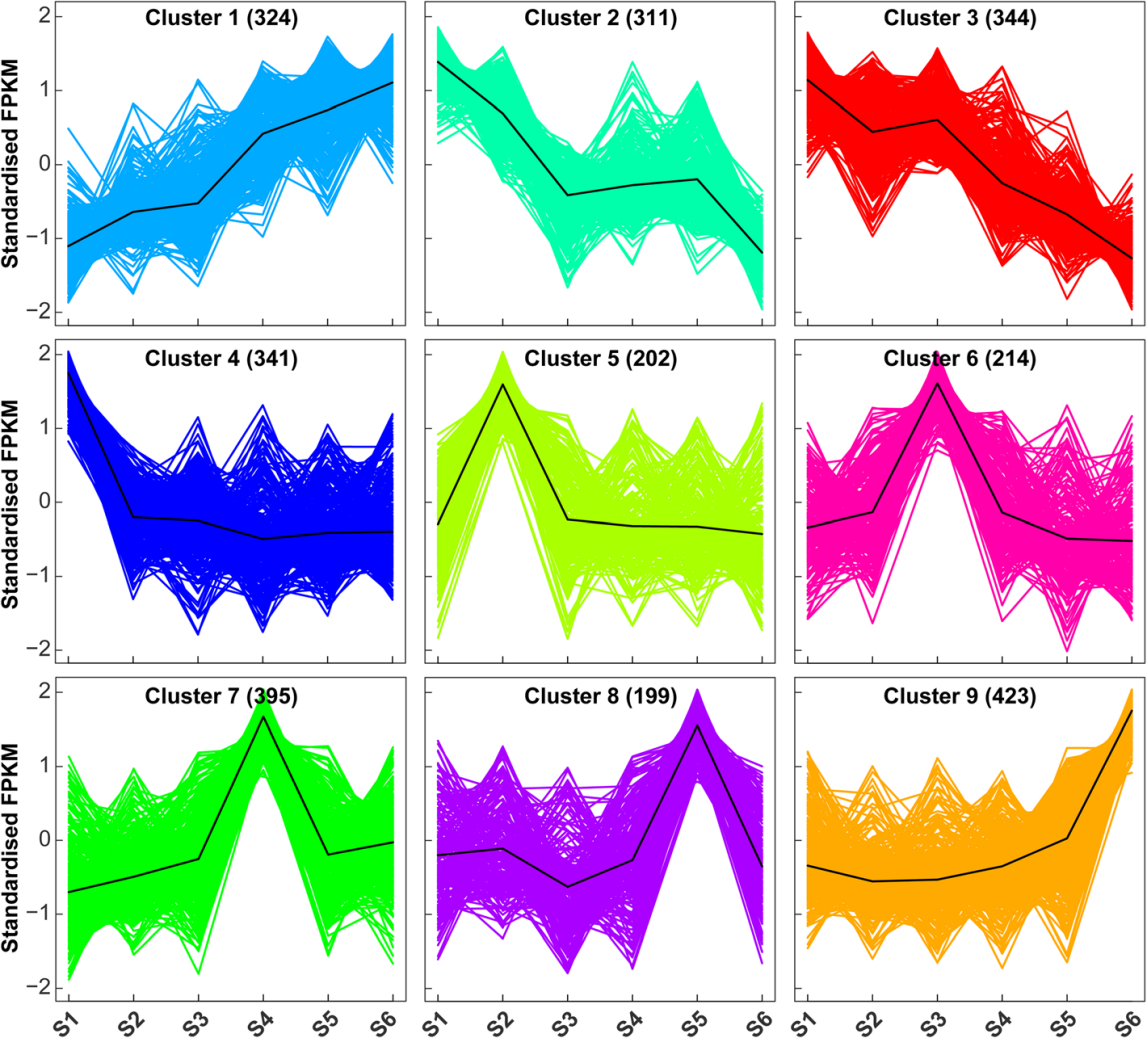
Clustering of differentially expressed lncRNAs. Cluster 1 represents the lncRNAs with consistently elevated expression from S1 to S6. Cluster 2 and 3 contain the lncRNAs with consistently decreased expression from S1 to S6 with varied patterns. Clusters 4–9 represent the lncRNAs that are abundantly expressed at a specific developmental stage among S1-S6, respectively. Numbers in the brackets represent numbers of lncRNAs in the corresponding clusters. The scaled FPKM value of all the differentially expressed lncRNA (2,753) was subjected to *k*-means clustering by Euclidean distance (*k* = 9)

To explore the possible regulation of lncRNAs during spike development, we next focused on the lncRNAs that were differentially expressed among the six developmental stages. Through pair-wise differential analysis by DEseq2 [27], a total of 2,753 DE lncRNAs with at least two-fold change in expression and a *p*-value less than 0.05 were identified (Supplementary Table S6). The numbers of DE lncRNAs between the earlier stages (S1-S3) were relatively less than those between the earlier stage (S1-S3) and later stage (S5-S6) (Supplementary Fig. S3), consistent with the progressive complexity of spike architecture during development. Using *k*-means clustering, we divided all DE lncRNAs into nine expression clusters (Fig. 2; Supplementary Table S6). The 324 lncRNAs in cluster 1 were those whose expressions were consistently elevated from S1 to S6, while 655 lncRNAs in clusters 2 and 3 included those whose expressions were consistently decreased from S1 to S6 with varied expression trends (Fig. 2). These clusters largely represented the lncRNAs that might function as positive or negative regulators during whole spike developmental progression. In contrast, the clusters 4-9 represented the lncRNAs that were abundantly expressed at a specific developmental stage among S1-S6 (Fig. 2). These lncRNAs likely participate in regulation of the distinct molecular events occurred at specific developmental stage. Further qRT-PCR analysis of six selected lncRNAs predominantly expressed at each developmental stage, including *MSTRG.60752*, *MSTRG.38633*, *MSTRG.2756*, *MSTRG.34036*, *MSTRG.11486*, and *MSTRG.39458*, validated the high reliability of their expression trends revealed by transcriptome data (Supplementary Fig. S4). In addition, we also amplified four lncRNAs, *MSTRG.59353*, *MSTRG.60752*, *MSTRG.38633* and *MSTRG.11486*, and validated their identity and expression through sanger sequencing.

### The coding genes potentially targeted by DE lncRNAs

**Fig. 3:**
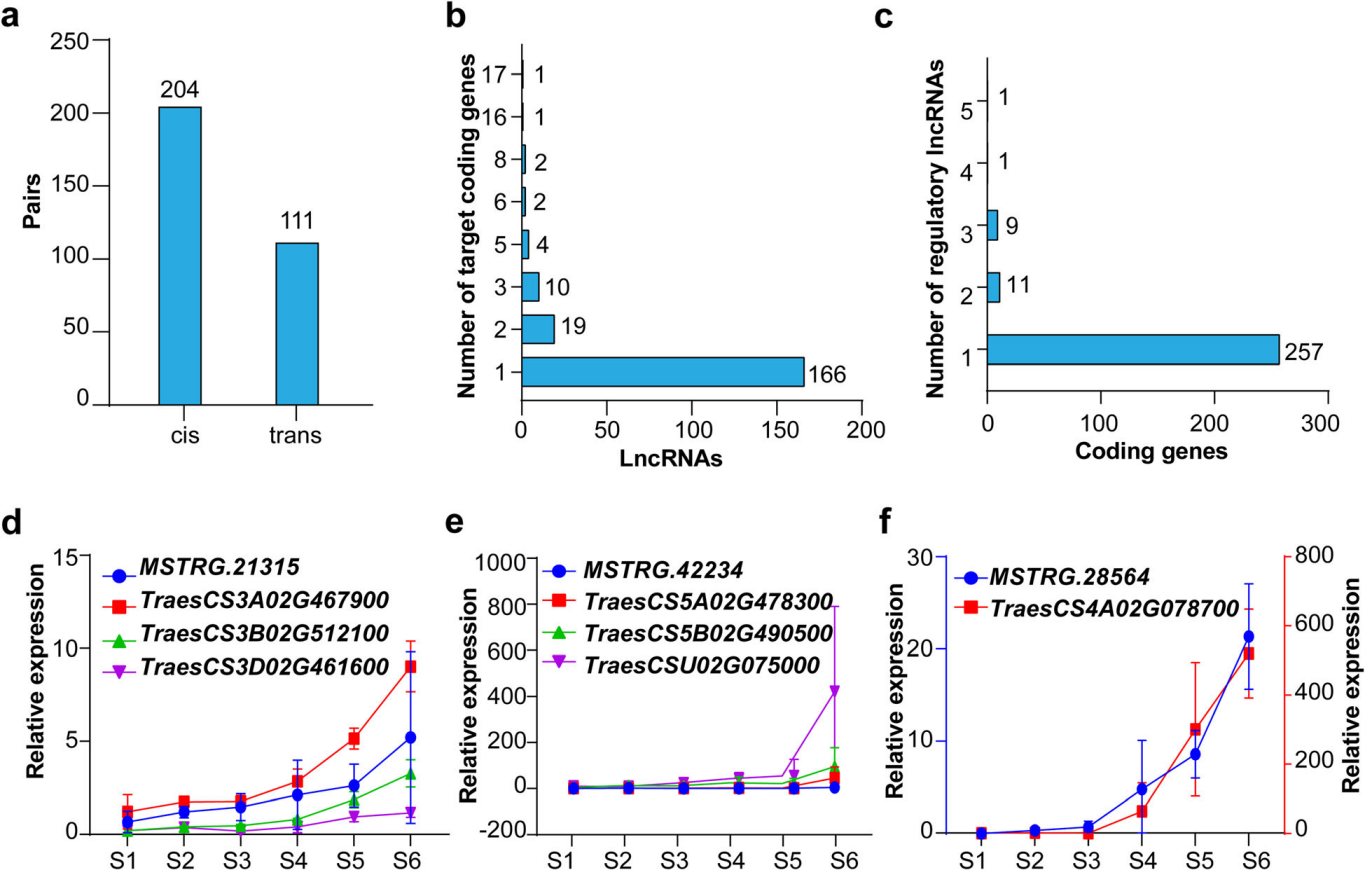
Predicted DE lncRNA-mRNA pairs and qRT-PCR verification of three regulatory pairs. **a** The number of identified *cis*- and *trans*-regulatory DE lncRNA-mRNA pairs. **b** Distribution of the number of target coding genes regulated by lncRNAs. **c** Distribution of the number of lncRNAs that have potential regulatory effects on coding genes. **d** Relative expression of *MSTRG.21315* and its three potential targets from S1 to S6. **e** Relative expression of *MSTRG.42234* and its three possible target genes. **f** Relative expression of *MSTRG.28564* and its potential target coding gene. The wheat *ACTIN* was used as an internal reference to normalize the qRT-PCR results. Data are from three biological replicates, and error bars indicate SD

Since lncRNAs can regulate gene expression either in *cis* or *trans* manner, we next analyzed the differentially expressed coding genes (DEGs) potentially targeted by DE lncRNAs based on the position and sequence relationships as well as their expression correlation coefficients. The lncRNAs and their potential target coding genes were thus referred as DE lncRNA-mRNA pairs. A total of 315 DE lncRNA-mRNA pairs composed of 205 lncRNAs and 279 coding genes were identified, of which 204 pairs were predicted to be *cis*-regulatory and 111 were *trans*-regulatory (Fig. 3a; Supplementary Table S7). Interestingly, all these lncRNA-mRNA pairs had a positive correlation in their expression patterns (Supplementary Table S7). Among these regulatory pairs, more than 80 % of lncRNAs (166) targeted the individual coding gene, while 29 lncRNAs targeted two or three genes, and ten lncRNAs had more than five target genes (Fig. 3b). Likewise, more than 92 % of coding genes (257) corresponded to an individual lncRNA, while 20 genes were targeted by two or three lncRNAs, and only two genes were targeted by four and five lncRNAs (Fig. 3c). These observations implicate that the regulatory crosstalk occurs between lncRNAs and coding genes. Further qRT-PCR analyses of three representative lncRNAs, *MSTRG.21315*, *MSTRG.42234*, *MSTRG.28564* and their predicted targets verified their expression correlations during spike development (Fig. 3d-f).

### GO enrichment analysis of coding genes regulated by DE lncRNAs

**Fig. 4:**
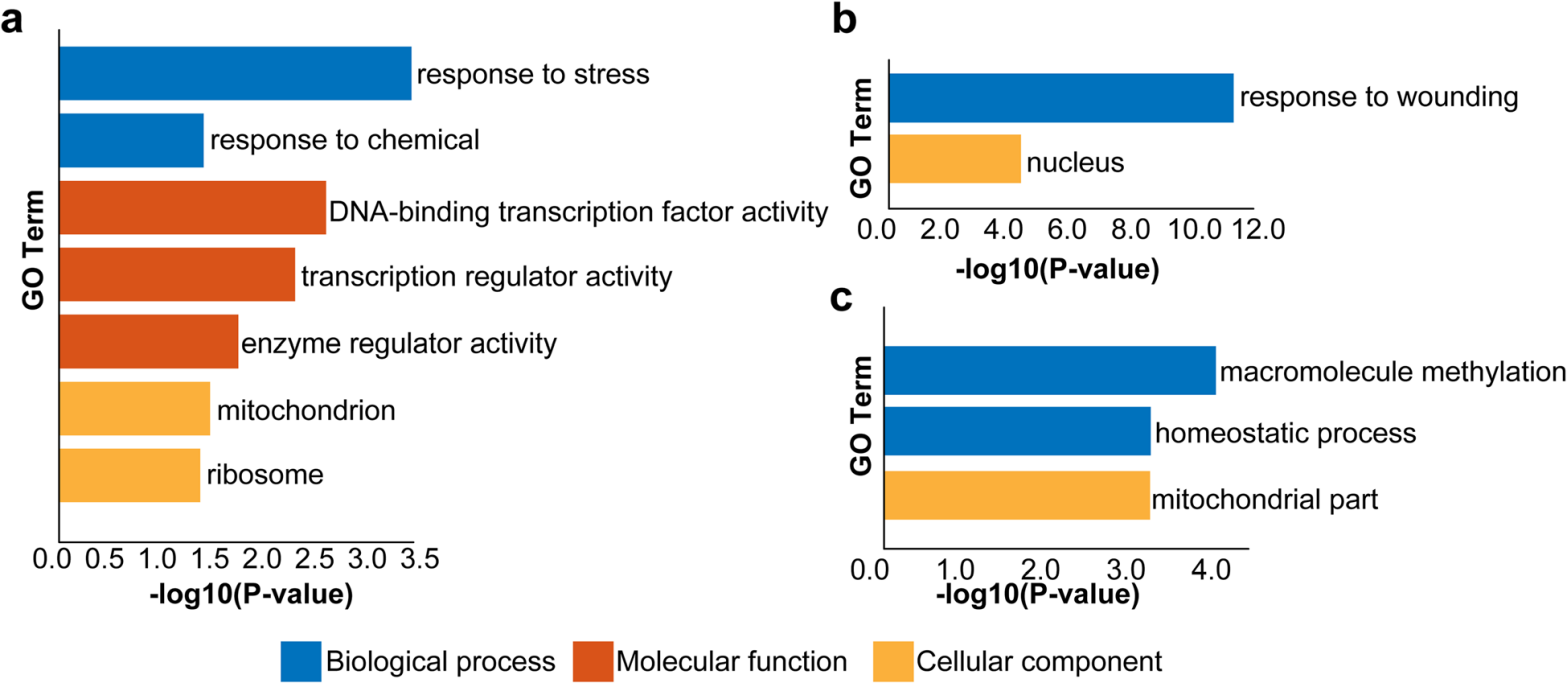
GO enrichment of the DEGs potentially targeted by DE lncRNAs. **a** GO enrichment of all 279 coding genes targeted by DE lncRNAs in *cis*- or *trans*- regulatory manner. **b** GO enrichment of DEGs potentially targeted by the lncRNAs with consistently elevated expression from S1 to S6. **c** GO enrichment of DEGs potentially targeted by the lncRNAs with consistently decreased expression from S1 to S6. GO was performed with three main categories: biological process, molecular function, and cellular component. GO terms with *P* value < 0.5 were identified as significant

To explore the molecular events regulated by DE lncRNAs, we performed Gene Ontology (GO) analysis of their target coding genes. Interestingly, among all the DEGs potentially targeted by DE lncRNAs, the genes associated with “response to stress” and “response to chemical” were highly enriched in biological process, and the genes associated with “transcription regulator activity” and “enzyme activity” were enriched in molecular function (Fig. 4a). Consistent with this, GO enrichment analysis of coding genes targeted by lncRNAs with gradually increased expression during spike development in cluster 1 further revealed that genes related to “response to wounding” and “nucleus” were enriched (Fig. 4b), and that genes associated with “macromolecule methylation” and “homeostatic process” were enriched among the DEGs targeted by lncRNAs with gradually decreased expression in cluster 2 and 3 (Fig. 4c). Therefore, the regulatory roles of lncRNAs during wheat spike development seemed to be mainly associated with stress response, transcriptional and enzymatic regulation.

**Fig. 5:**
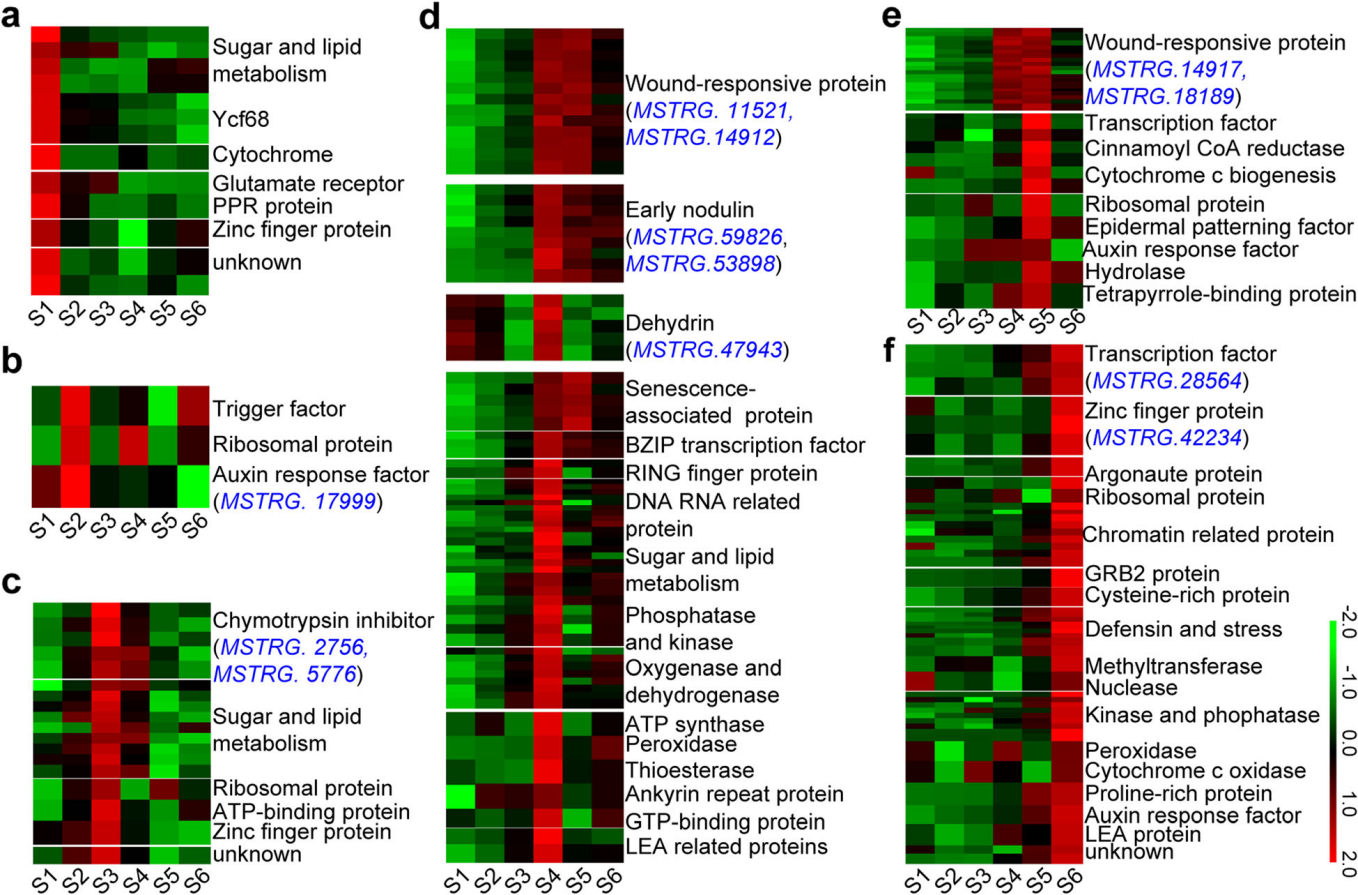
DEGs targeted by stage-specific lncRNAs. **a-f** Dynamic expression of DEGs potentially targeted by the lncRNAs predominantly expressed at the six developmental stage, respectively. The important lncRNAs involved in the indicated biological processes are shown in the following brackets with blue color. The normalized expression levels of DEGs at six developmental stages were used to generate heatmaps by TBtools

As the coding genes targeted by stage-specific lncRNAs might contribute to the spike patterning at an individual developmental stage, we also examined the genes targeted by lncRNAs in clusters 4–9 (Fig. 5; Supplementary Table S8). As expected, these coding genes indeed exhibited predominant expressions at the corresponding developmental stage as did their paired lncRNAs (Fig. 5). At S1, there were four genes encoding metabolic related proteins, three encoding Ycf68 proteins with unknown functions, several genes encoding cytochrome, glutamate receptor, pentatricopeptide repeat-containing protein and zinc finger protein (Fig. 5a). At S2, three lncRNAs were involved in regulation of three genes encoding a trigger factor, a ribosomal protein, and an auxin response factor (Fig. 5b). At S3, the genes encoding chymotrypsin inhibitors, and sugar or lipid metabolism related proteins were identified as potential targets of lncRNAs (Fig. 5c). Interestingly, a large portion of genes encoding wound-responsive proteins, dehydrin proteins and early nodulin related proteins were found to be targeted by lncRNAs at S4 (Fig. 5d). Similarly, among 33 genes targeted by lncRNAs at S5, there were 22 genes that encode wound-responsive proteins (Fig. 5e; Supplementary Table S8). This observation implicates that lncRNAs-regulated stress response especially wounding response may contribute to floret, stamen and pistil primordium formation at S4 and S5. Among genes potentially targeted by lncRNAs at S6, there were several genes encoding MADS-box TFs, zinc finger proteins and Argonaute1 proteins, and a large portion of genes were found to encode nuclear proteins, such as chromatin related proteins (Fig. 5f), implying that the transcription regulation and epigenetic modification regulated by lncRNAs are critical for floral organ development.

### Identification of important lncRNAs involved in wheat spike development

To identify the important lncRNAs involved in spike development, we closely examined the DE lncRNAs related with stress response, TFs regulation, hormone signaling and chromatin modeling (Table 1; Fig. 5). Among DE lncRNAs related to stress response, there were six lncRNAs, including *MSTRG.18189*, *MSTRG.18191*, *MSTRG.14917*, *MSTRG.11521*, *MSTRG.14912* and *MSTRG.11500*, that potentially targeted a total of 30 wound-responsive genes (Table 1), while *MSTRG.59826* and *MSTRG.53898* were found to target nine nodulin-related genes, and *MSTRG.47943* targeting five dehydrin genes (Table 1; Fig. 5). By contrast, there were 12 TF genes identified in the DE lncRNA-mRNA pairs, including two MADS-box, eight bZIP and two MYB genes (Table 1). Noticeably, *MSTRG.28564* and *MSTRG.61765* potentially targeted two MADS-box genes, *TraesCS4A02G078700* and *TraesCS7D02G380300*, while *MSTRG.51463*, *MSTRG.62263*, *MSTRG.39593* and *MSTRG.43002* potentially targeted eight bZIP genes, and *MSTRG.57306*, *MSTRG.30797* targeted two MYB genes (Table 1). Moreover, auxin has been reported to play critical roles during inflorescence development [13], we also observed that four auxin response genes, including *TraesCS2D02G491200*, a homolog of *AUXIN RESPONSE FACTOR 5* (*ARF5*) in *Arabidopsis*, were targeted by four lncRNAs (Table 1). In addition, Argonaute1 (AGO1) can bind to chromatin to regulate transcription in association with SWI/SNF complexes [28]. We also found that *MSTRG.59353* could target *AGO1d-7A* and *AGO1d-7B*, and that *MSTRG.23144* and *MSTRG.19854* possibly targeted two SWI/SNF-related genes (Table 1). These DE lncRNAs and their target genes likely represent the key regulatory networks of lncRNAs during wheat spike patterning and development.

**Table 1:** The identified important lncRNAs and their targeted coding genes involved in wheat spike development

LncRNA ID	*k*-means cluster	Target gene ID	Description
*MSTRG.18189*	8	*TraesCS2D02G534200, TraesCS2D02G533500, TraesCS2D02G533600, TraesCS2D02G533700, TraesCS2D02G533800, TraesCS2D02G533900, TraesCS2D02G534100, TraesCS2D02G534500, TraesCS2B02G561000, TraesCS2A02G532000, TraesCS2A02G531800, TraesCS2D02G533100, TraesCS2B02G513900, TraesCS2A02G532200, TraesCS2B02G560900, TraesCS2D02G487100, TraesCS2B02G513800*	Wound-responsive family protein
*MSTRG.18191*	1	*TraesCS2D02G534200, TraesCS2D02G533700, TraesCS2D02G533800, TraesCS2D02G534100, TraesCS2D02G534500, TraesCS2D02G533400, TraesCS2D02G533500, TraesCS2A02G531800, TraesCS6B02G162700, TraesCS2D02G533100, TraesCS2A02G532100, TraesCS2B02G513900, TraesCS2A02G532200, TraesCS2D02G532000, TraesCS2D02G487100, TraesCS2B02G561200*	Wound-responsive family protein
*MSTRG.11500*	1	*TraesCS2B02G561000, TraesCS2A02G531800, TraesCS2A02G532100, TraesCS2B02G513900, TraesCS2D02G533800, TraesCS2B02G560900, TraesCS2B02G561300, TraesCS2B02G561400*	Wound-responsive family protein
*MSTRG.11521*	7	*TraesCS2B02G561000, TraesCS2D02G534200, TraesCS2A02G532000, TraesCS2D02G533500, TraesCS2A02G531800, TraesCS2D02G533100, TraesCS2A02G532200, TraesCS2D02G487100*	Wound-responsive family protein
*MSTRG.14912*	7	*TraesCS2B02G561300, TraesCS2B02G561400, TraesCS2B02G561500, TraesCS2A02G531800, TraesCS2D02G533800*	Wound-responsive family protein
*MSTRG.14917*	8	*TraesCS2B02G561900, TraesCS2B02G562000, TraesCS2B02G561600, TraesCS2A02G532300, TraesCS2D02G534100*	Wound-responsive family protein
*MSTRG.59826*	7	*TraesCS7D02G087600, TraesCS7D02G087700, TraesCS7D02G088000, TraesCS7D02G088100, TraesCS7D02G087500, TraesCS7D02G087800*	Early nodulin 93
*MSTRG.53898*	7	*TraesCS7A02G092000, TraesCS7A02G093100, TraesCS7D02G088600*	Early nodulin 93
*MSTRG.47943*	7	*TraesCS6A02G350100, TraesCS6B02G383200, TraesCS6A02G350300, TraesCS6D02G332500, TraesCS6D02G332700*	Dehydrin
*MSTRG.28564*	9	*TraesCS4A02G078700 (MADS4)*	MADS box transcription factor
*MSTRG.61765*	9	*TraesCS7D02G380300 (MADS16)*	MADS box transcription factor
*MSTRG.51463*	3	*TraesCS6B02G124700, TraesCS6A02G096300, TraesCS6D02G087400*	BZIP transcription factor
*MSTRG.62263*	7	*TraesCS7A02G488600, TraesCS7B02G391800, TraesCS7D02G475100*	BZIP transcription factor
*MSTRG.39593*	8	*TraesCS5B02G059200*	BZIP transcription factor
*MSTRG.43002*	8	*TraesCS5D02G068800*	BZIP transcription factor
*MSTRG.57306*	9	*TraesCS7B02G131600*	Transcription factor RADIALIS
*MSTRG.30797*	8	*TraesCS4A02G474100*	MYB-related transcription factor
*MSTRG.29168*	1	*TraesCS4A02G158100*	Auxin-repressed protein
*MSTRG.32137*	8	*TraesCS4B02G161800*	Auxin response factor
*MSTRG.51533*	9	*TraesCS6D02G100900*	Auxin response factor
*MSTRG.17999*	5	*TraesCS2D02G491200 (ARF5)*	Auxin response factor
*MSTRG.59353*	9	*TraesCS7A02G557400, TraesCS7B02G482100 (AGO1d)*	Protein Argonaute
*MSTRG.23144*	9	*TraesCS3B02G260400*	SWI/SNF-related protein
*MSTRG.19854*	9	*TraesCS3A02G231100*	SWI/SNF-related protein

### LncRNAs as putative miRNA precursors or target mimics

**Fig. 6:**
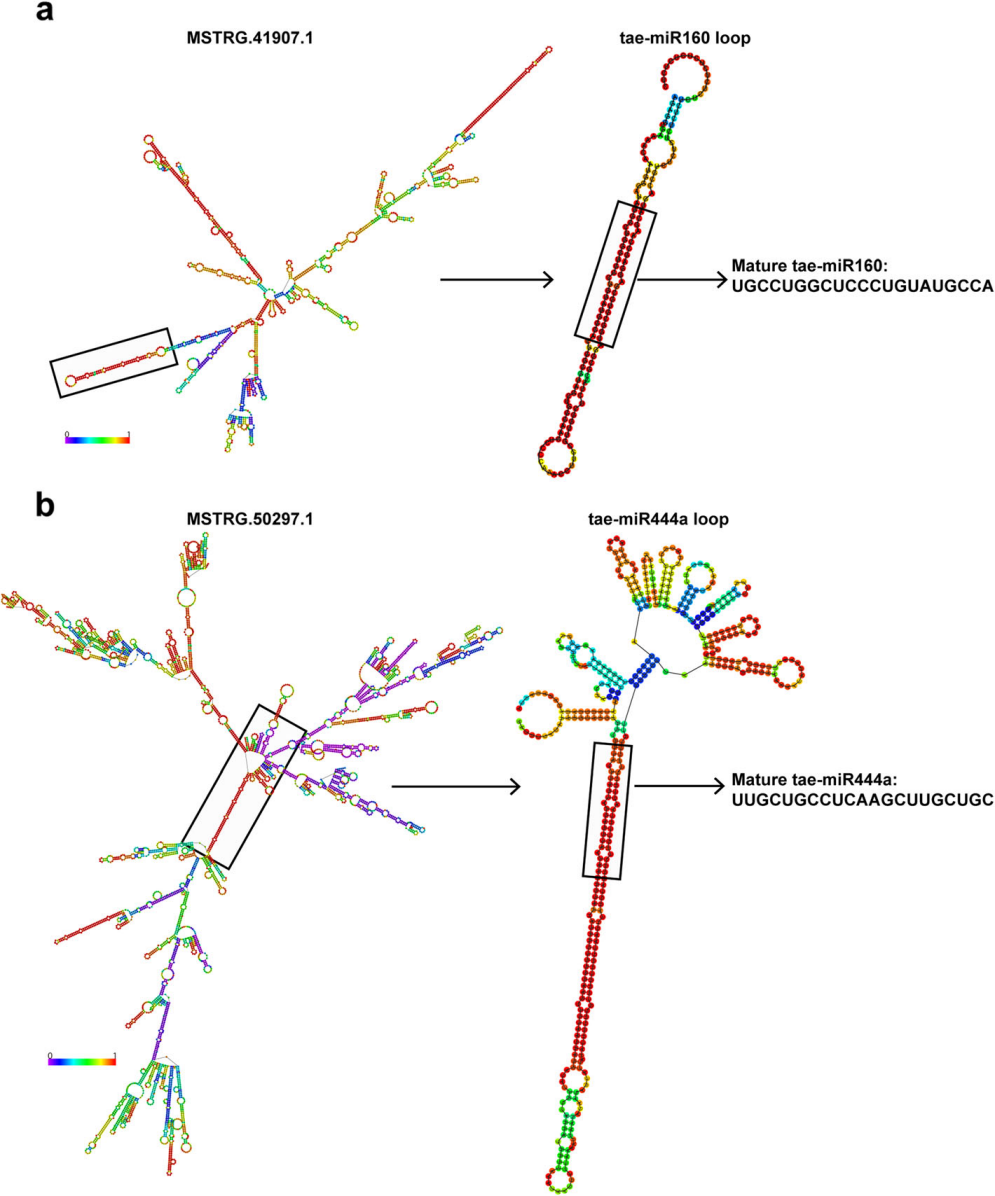
Predicted structures of two representative lncRNAs and their corresponding miRNA precursors. **a** Structure of *MSTRG.41907.1*, stem-loop of tae-miR160, and matured tae-miR160. **b** Structure of *MSTRG.50297.1*, stem-loop of tae-miR444a, and matured tae-miR444a

LncRNAs can also act as precursors or target mimics of miRNA to regulate miRNAs’ function, and some of miRNAs have been reported to be involved in wheat spike development, such as miR172 and miR156 [29–31]. To identify the lncRNAs that possibly act as precursors or target mimics of miRNA, we further performed sequence alignment to miRBase and psRNAtarget [32, 33]. A total of 58 DE lncRNAs were predicted as putative precursors of 21 miRNAs, including tae-miR156, tae-miR160, tae-miR167 and tae-miR444 (Supplementary Table S9). We also observed that one lncRNA might serve as the precursors for one or a few of miRNAs, while several lncRNAs might be potential precursors for the same miRNA (Supplementary Table S9). Notably, *MSTRG.41996* and *MSTRG.45365* that were highly expressed at S1 and S2 were predicted as the precursors of tae-miR167a and tae-miR167b, while *MSTRG.41907* highly expressed during S1-S3 was predicted as the precursor of tae-miR160, and *MSTRG.50297* predominantly expressed at S1 and S6 was predicted as the precursor of tae-miR444a (Supplementary Table S6, S9). To further verify this, we used RNAfold web server (http://rna.tbi.univie.ac.at/cgi-bin/RNAWebSuite/RNAfold.cgi) to predict the secondary structures of the two representative lncRNAs and their miRNA precursors. As shown in Fig. 6a, the secondary structure of *MSTRG.41907.1* exhibited multiple stem-loop structures, one of which could be cleaved to release the precursor sequence of tae-miR160 and eventually form mature tae-miR160. Similarly, the secondary structure of *MSTRG.50297.1* might be cleaved to produce precursor of tae-miR444a and form mature tae-miR444a (Fig. 6b). Furthermore, transcripts from 87 DE lncRNAs were predicted as target mimics of 37 miRNAs, including tae-miR1139, tae-miR9672, tae-miR9674, tae-miR9783 and other miRNAs (Supplementary Table S10). These observations support that these lncRNAs participate in wheat spike development as precursors or target mimics of miRNA.

## Discussion

**Fig. 7:**
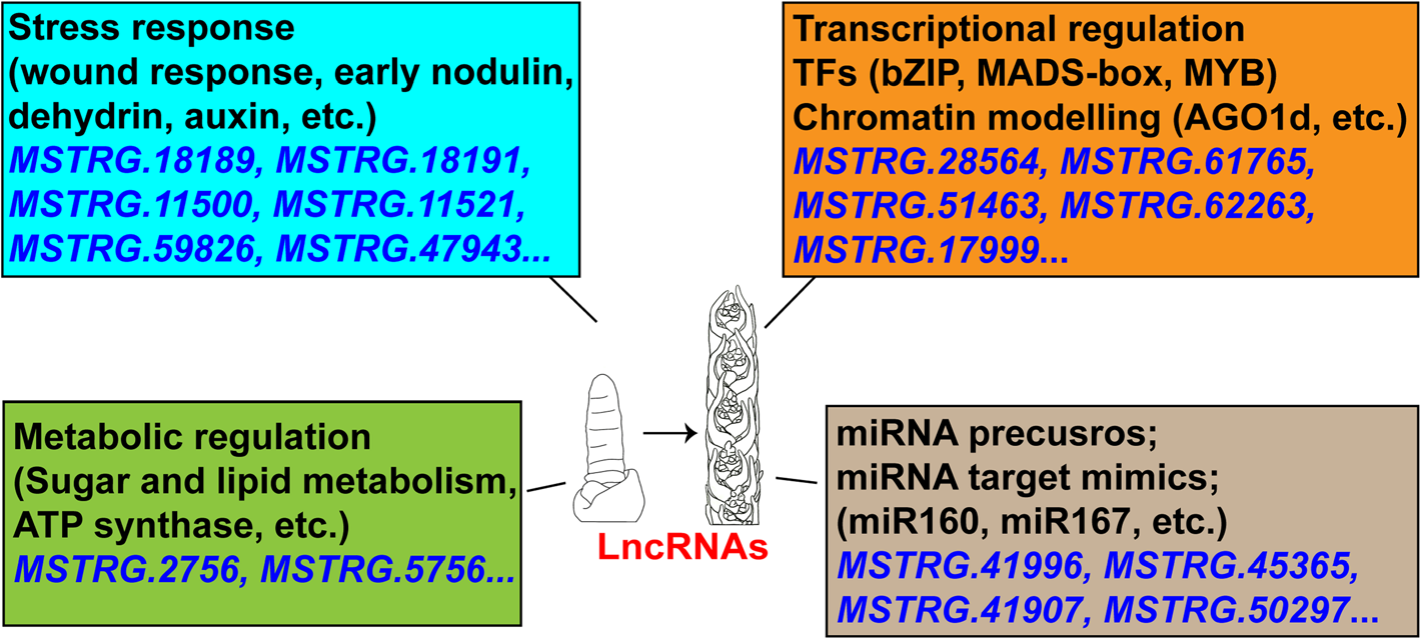
The molecular regulation of lncRNAs during wheat spike development. LncRNAs participate in wheat spike development mainly by targeting the coding genes associated in stress responses (wound, nodulin, dehydrin, auxin, etc.), transcriptional regulation (TFs, chromatin, etc.), and metabolic regulation (sugar and lipid, etc.). Some lncRNAs act as potential precursors or target mimics of miRNAs, such as miR160, miR167, and thus influence spike development

LncRNAs have been reported to play important roles in plant growth and development in various plant species [18, 20–22]. Recent works have also identified a series of lncRNAs during wheat grain development, tillering development, and defense-response [25, 34, 35]. However, lncRNAs and their possible regulations during wheat spike development remain elusive. Here, we monitored the genome-wide profiling of lncRNAs and identified a total of 8,889 lncRNAs expressed in wheat spike, of which 2,753 lncRNAs are differentially expressed among six developmental stages. We predicted 315 DE lncRNA-mRNA regulatory pairs and 145 potential miRNA precursors or target mimics, and outlined the possible molecular events regulated by lncRNAs (Fig. 7). Our work provides a glimpse of lncRNAs and their molecular regulations during wheat SM, FM and floral organ development. As wheat spike development is critical for determination of agronomic traits such as spikelet, floret and grain numbers, further works on these lncRNAs and their potential targets will be necessary not only for understanding the lncRNAs and their molecular regulations during wheat spike development, but also for genetic modification of spike architecture and thus wheat yield.

Since lncRNAs can execute their functions by *cis-* or *trans-*regulation of target coding genes [15, 16], the identification of their possible target genes is necessary for understanding their regulatory roles. Interestingly, our GO enrichment of genes targeted by DE lncRNAs revealed that these genes were mainly associated with stress response, transcription regulation and enzyme activity regulation (Fig. 4), suggesting that lncRNAs may mainly target on these biological processes to regulate spike development. Indeed, stress responses in plants, such as drought, cold, hormone and so on, not only influence wheat spikelet and grain numbers and thus yield under ever-changed environments [36–38], but also play important roles in the cell fate determination during plant meristem differentiation and regeneration [39, 40]. For example, wounding is one of key signals to trigger cell reprograming in plant regeneration, and the following auxin, jasmonic acid, ethylene, and electrical pulses are critical for wound-triggered cell fate switches [41, 42]. As wheat spike formation and development involve multiple meristem differentiation and organ formation, it is likely that lncRNAs may participate in regulation of such meristem differentiation by targeting the stress and hormone related genes. Consistent with this, four genes encoding auxin response factors were regulated by four lncRNAs with various expression patterns (Table 1). Meanwhile, a total of 134 and 27 DEGs related with auxin and cytokinin were also detected during spike development, respectively (Supplementary Table S3). On the other hand, TFs, such as AP2, SPL, bZIP and MADS-box, have been reported to play essential roles during inflorescence development in wheat and other plants [5, 6, 10, 30]. Here, we found that 12 TF genes were potentially targeted by lncRNAs (Table 1), suggesting that these lncRNAs may also facilitate their roles on transcriptional regulation of key TFs required for wheat spike development. In addition, metabolic process influences spike and grain numbers in crops mainly through accumulation of assimilates, especially for carbohydrates, which is controlled by a series of enzymes [43–46]. The enrichment of enzyme-related genes demonstrates that cellular metabolic processes essential for spike organs (spikelet, glume and floret) development are also regulated by lncRNAs.

As wheat spike development undergoes subsequent developmental processes of multiple meristems and floral organs (S1-S6), identification of the stage-specific lncRNAs and their target genes will be helpful to understand the specific events for spike patterning regulated by lncRNAs. We observed that some of metabolic related genes targeted by lncRNAs were mainly enriched during S1-S4 (Fig. 5; Supplementary Table S8), suggesting that the regulation of these metabolic processes by lncRNAs may contribute to the formation of various meristems (spikelet, glume, lemma, and floret) at early developmental stages. By contrast, the enrichment of a series of genes associated with wound responses by lncRNAs at S4 and S5 implicates that the floret, stamen and pistil primordium formation may require the precise regulations of lncRNAs during the cell fate changes and environmental responses at these stages (Fig. 5; Supplementary Table S8). Moreover, the enrichment of TF and chromatin-related genes targeted by lncRNAs at S5 and S6 demonstrates that lncRNAs may regulate floral organ development through transcriptional and epigenetic modification (Fig. 5; Supplementary Table S8). Finally, we identified 24 important lncRNAs that were involved in various regulations during wheat spike development and found some of them may mainly function at specific stage (Table 1). For examples, *MSTRG.18189*, *MSTRG.11521*, *MSTRG.14912* and *MSTRG.14917* could target a series of wound responsive genes at S4 and S5, and *MSTRG.59353* possibly target *AGO1d-7 A* and *AGO1d-7B* at S6 (Fig. 5; Table 1). As wheat *AGO1d* has been shown to play an important role in anther and pollen development [12], it is likely that *MSTRG.59353* may be involved in floret fertility development. Therefore, these important lncRNAs and their potential targets will be the candidates for further elucidating the molecular regulations of lncRNAs and for genetically modification of wheat spike architecture.

MiRNAs play important roles in regulating plant morphogenesis, including the transition of reproductive phase from vegetative phase and the establishment of organ patterning [47–50]. In wheat, several key miRNAs have been reported to participate in spike development. For examples, miRNA156 targets the *SPL* gene to regulate spikelet development, while miRNA172 modifies wheat spike architecture through *Q* gene [31, 51, 52]. Transcriptomic analysis also found that miR159, miR167, and miR319 were abundantly expressed in wheat inflorescence meristems [12]. Here, we also predicted some DE lncRNAs as putative precursors or target mimics of these important miRNAs, strengthening that these lncRNAs may facilitate their regulations in wheat spike development through modifying functional miRNAs and their target genes. For examples, *MSTRG.2923* may act as the precursor of tae-miR156 and tae-miR160, and *MSTRG.45365* as the precursor of tae-miR167 (Supplementary Table S9). We also identified some lncRNAs may function as precursors or targets of miRNAs with unknown functions, such as miR1135 and miR1136. Further works on these lncRNAs and their corresponding miRNAs will be helpful to clarify their molecular interactions during wheat spike development.

## Conclusions

In this study, a total of 8,889 lncRNAs are identified to be expressed during wheat spike development, among which 2,753 DE lncRNAs are categorized into nine expression clusters. A total of 315 DE lncRNA-mRNA *cis*- and *trans*-regulatory pairs are predicted, among which 24 important lncRNAs and their regulatory genes are identified. They are mainly related to stress responses, transcriptional regulation, and enzyme activity regulation. Moreover, 58 DE lncRNAs are identified as potential miRNA precursors and 87 DE lncRNAs as miRNA target mimics. These findings provide an overall view on lncRNAs and their possible regulatory networks in wheat spike development.

## Methods

### Plant materials and sample collection

The winter wheat cultivar Zhengmai366, a widely bred cultivar for high-yield and good-quality wheat, is obtained from the breeder of Wheat Research Institute, Henan Academy of Agricultural Sciences, with permissions for usage and collection of plants and seeds. All the plant growth management and methods used in this study are complied with relevant national guidelines and legislations of China (http://www.moa.gov.cn). The Zhengmai366 was grown in Beijing, China and the developmental stages of spikes were determined based on the anatomic and morphological features under stereomicroscope OLYMPUS SZX16 (Japan) according to previous study [8]. The developing spikes at six developmental stages referred as S1 to S6 were collected, which represented the developmental stages of IM, SM, glume primordium, FM, stamen and pistil primordia, and floral organs (anther and awn), respectively. The dissected spikes were immediately frozen in liquid nitrogen and stored at -80 °C. About 20-50 spikes at each developmental stage were pooled for each of the three biological replicates and subjected for RNA isolation and sequencing.

### Scanning electron microscopy

To determine the developmental stage, the developing spikes were fixed in FAA (50 % v/v ethanol, 5 % v/v acetic acid, and 3.7 % v/v formaldehyde) for 24 h at 4 °C, dehydrated through a standard ethanol series, and dried with CO_2_ critical point. The dried samples were mounted on stubs, coated with gold, and photographed with a Hitachi S-4800 device (Tokyo, Japan).

### RNA isolation, library construction and sequencing

Total RNA was isolated with TRIzol reagent (Invitrogen) and treated with DNaseI (Invitrogen) to eliminate contaminating DNA. Construction of transcriptome libraries and deep sequencing were performed by The Beijing Genomics Institute. Ribosomal RNA was removed using a Ribo-Zero™ rRNA Removal Kit (Illumina). Subsequently, random primers from TruSeq® Stranded Kit (Illumina) were used to synthesize cDNA with the templates of fragmented RNAs. The cDNA library was obtained by PCR amplification. The resulting libraries were sequenced on a HiSeq 4000 instrument (Illumina) that generated paired end reads of 100 bp.

### Reads mapping, transcript assembly and lncRNAs identification

After removing the adaptor-polluted and low-quality reads, approximately 15 Gb clean base pairs for each sample were mapped to the *Triticum aestivum* L. reference genome assembly (IWGSC Refseq v1.0, Ensemble Plants, http://ftp.ensemblgenomes.org/pub/plants/release-46/) using HISAT with default parameters [53]. Stringtie was used to assemble the transcripts and Gffcompare was used to compare these transcripts with known gene annotation models to predict novel transcripts [54]. The protein-coding capacity of novel transcripts with length of > 200 bp was identified using LGC and CNCI with default parameters, respectively [55]. Then Pfam database was used to ensure the predicted transcripts have no protein-coding domains [56]. The transcripts as lncRNAs were determined by consistency of the three judgement methods.

### Expression analysis of lncRNAs

FPKM were calculated to estimate the expression level of genes in each sample. The lncRNAs and coding genes with an average FPKM of ≥ 1 in at least one stage were considered as expressed. TPM were calculated to compare the gene abundance levels in the same stage. Circos software was used to visually describe the distribution of expressed lncRNAs on 21 chromosomes [57]. DEseq2 was used to perform differential expression analysis among various developmental stages [27]. DE lncRNAs and DEGs were filtered with at least two-fold expression change and a *p*-value less than 0.05. Clustering of DE lncRNAs was performed by R version 3.6.2 using *k*-means function. Heat maps and the Venn diagram were drawn using TBtools [58].

### Validation of selected lncRNAs by qRT-PCR and Sanger sequencing

Total RNA was isolated with TRIzol reagent (Invitrogen). Reverse transcription was performed using the PrimeScript RT reagent Kit with gDNA Eraser following the manufacturer’s recommendations (Takara). Real-time quantitative PCR was performed using the SYBR Premix Ex TaqTM Kit (Takara) on a LightCycler 96 machine (Roche). The wheat *ACTIN* gene was used as a reference control and relative expression levels were calculated using the 2^-▵▵CT^ method [59]. The qRT-PCR was performed with three biological replicates and all the primers sequence information was listed in Supplementary Table S11. Four lncRNAs were amplified using the Phusion high-fidelity enzyme (NEB) and sequenced by Sanger sequencing in The Beijing Genomics Institute.

### Prediction of lncRNA target genes and GO analysis

The lncRNA and its predicted *cis-* or *trans-*regulatory coding genes were considered as lncRNA-mRNA pairs. The *cis*-regulatory genes of lncRNA were determined if there was an overlap between the lncRNA and the coding genes, or the lncRNA located within 100 kb up- or down-stream of the coding genes [60]. For *trans*-regulation, LncTar was used to calculate the free energy of lncRNA-mRNA pairs and the normalized free energy at -0.1 was set as cutoff to determine target genes [61]. In addition, the lncRNA-mRNA pairs were filtered with the Pearson correlation efficient of their expression levels (|r|> 0.9). The DEGs possibly targeted by DE lncRNAs were considered as candidate coding genes of interest for further analysis. GO enrichment analysis was performed with TBtools software. The GO terms with a *p*-value below 0.05 were considered significantly enriched.

### Prediction of miRNA precursors or target mimics

To identify potential miRNA precursors, lncRNAs were blasted to miRBase (http://www.mirbase.org/search.shtml) and the identity between lncRNAs and miRNA precursors that were 100 % and e-value < 0.001 were selected [32]. The miRNA target mimics prediction was performed by aligning the wheat mature miRNA sequences against lncRNA sequences using psRNAtarget with default parameters except for a strict Expectation value 2 [33].

## Supplementary Information

**Additional file 1: Supplementary Figure S1** Morphology of wheat spike at six developmental stages. **a-f** Morphology of spikes at the six developmental stages (S1-S6) under a stereomicroscope. **g-l** The scanning electron microscopy images of the spikes described in a-f. SM, spikelet meristem. GP, glume primordium. LP, lemma primordium. FM, floret meristem. STP, stamen primordium. PP, pistil primordium. ANP, anther primordium. AWP, awn primordium. Bars: a-f, 1mm; g, 0.1 mm; h, 0.2 mm; i, 0.3 mm; j, 0.4 mm; k, 0.3 mm; l, 0.5 mm. **Supplementary Figure S2** Venn diagrams of the lncRNAs expressed in wheat spike. Six developmental stages (S1 to S6) are indicated by different colors. **Supplementary Figure S3** Numbers of DE lncRNAs among developmental stages. The numbers of DE lncRNAs with increased or decreased expression between two compared stages are shown in red or blue, respectively. DE lncRNAs were filtered according to *p*-value < 0.05 and log_2_ (fold change) > 1 or < − 1. **Supplementary Figure S4** Quantitative RT-PCR analysis of six representative lncRNAs. The expression levels of six representative lncRNAs are shown based on the data from qRT-PCR (blue) and RNAseq (red), respectively. The wheat *ACTIN* was used as an internal reference to normalize the qRT-PCR results. Data are from three biological replicates and error bars indicate SD.

**Additional file 2: Supplementary Table S1** RNA-seq reads and genome mapping rates. **Supplementary Table S2** Coding genes identified in wheat spike. **Supplementary Table S3** Differentially expressed coding genes among six developmental stages. **Supplementary Table S4** LncRNAs identified in wheat spike. **Supplementary Table S5** LncRNAs expressed at individual developmental stage. **Supplementary Table S6** Expression level and *k*-means clustering assignment of differentially expressed lncRNAs. **Supplementary Table S7** DE lncRNA-mRNA pairs in *cis-* or *trans*-regulatory mode. **Supplementary Table S8** Stage-specific lncRNAs and their target coding genes. **Supplementary Table S9** LncRNAs as putative MiRNA precursors. **Supplementary Table S10** LncRNAs as putative miRNA target mimics. **Supplementary Table S11** Primers used in this study.

## Data Availability

The raw sequence reads are available in NCBI sequence read archive database and can be accessed under the ID PRJNA718695 (https://www.ncbi.nlm.nih.gov/bioproject/PRJNA718695) (NCBI Sequence Read Archive SRR14116504, SRR14116505, SRR14116506, SRR14116507, SRR14116508, SRR14116509, SRR14116510, SRR14116511, SRR14116512, SRR14116513, SRR14116514, SRR14116515, SRR14116516, SRR14116517, SRR14116518, SRR14116519, SRR14116520 and SRR14116521). All other relevant data generated or analyzed in this study are included in this article and its Additional files.

## Abbreviations

DEGs: Differentially expressed coding genes
DE lncRNAs: Differentially expressed lncRNA
FM: Floret meristem
FPKM: Fragments per kilobase of exon model per million mapped reads
GO: Gene Ontology
IM: Inflorescence meristem
LncRNAs: Long noncoding RNAs
SAM: Shoot apical meristem
SM: Spikelet meristem
TPM: Transcripts per kilobase of exon model per million mapped reads
