# Supplementary material for: Genome-wide profiling of long noncoding RNAs involved in wheat spike development
Source: BMC Genomics. 2021 Jul 2;22:493. doi: 10.1186/s12864-021-07851-4 (PMC8252277; doi:10.1186/s12864-021-07851-4)
Supplement: Supplementary file 1 — Additional file 1: Supplementary Figure S1 Morphology of wheat spike at six developmental stages. a-f Morphology of spikes at the six developmental stages (S1-S6) under a stereomicroscope. g-l The scanning electron microscopy images of the spikes described in a-f. SM, spikelet meristem. GP, glume primordium. LP, lemma primordium. FM, floret meristem. STP, stamen primordium. PP, pistil primordium. ANP, anther primordium. AWP, awn primordium. Bars: a-f, 1mm; g, 0.1 mm; h, 0.2 mm; i, 0.3 mm; j, 0.4 mm; k, 0.3 mm; l, 0.5 mm. Supplementary Figure S2 Venn diagrams of the lncRNAs expressed in wheat spike. Six developmental stages (S1 to S6) are indicated by different colors. Supplementary Figure S3 Numbers of DE lncRNAs among developmental stages. The numbers of DE lncRNAs with increased or decreased expression between two compared stages are shown in red or blue, respectively. DE lncRNAs were filtered according to p-value < 0.05 and log2 (fold change) > 1 or < − 1. Supplementary Figure S4 Quantitative RT-PCR analysis of six representative lncRNAs. The expression levels of six representative lncRNAs are shown based on the data from qRT-PCR (blue) and RNAseq (red), respectively. The wheat ACTIN was used as an internal reference to normalize the qRT-PCR results. Data are from three biological replicates and error bars indicate SD. [file 12864_2021_7851_MOESM1_ESM.pdf]

Genome-wide profiling of long noncoding RNAs involved in wheat spike development

Pei Cao[1], Wenjuan Fan[1,2], Pengjia Li[1,2], Yuxin Hu[1,3,*]

[1] Key Laboratory of Plant Molecular Physiology, CAS Center for Excellence in Molecular Plant Sciences, Institute of Botany, Chinese Academy of Sciences, Beijing 100093, China

[2] University of Chinese Academy of Sciences, Beijing 100049, China

[3] National Center for Plant Gene Research, Beijing 100093, China

* Corresponding author: Yuxin Hu, Institute of Botany, Chinese Academy of Sciences, Beijing 100093, China. Email address: huyuxin@ibcas.ac.cn. Tel: +86-10-62836650. Fax: +86-10-62836691.

Table of Contents

Supplementary FigureS1: Morphology of wheat spike at six developmental stages.

Supplementary Figure S2: Venn diagrams of the lncRNAs expressed in wheat spike. Six developmental stages (S1 to S6) are indicated by different colors.

Supplementary Figure S3: Numbers of DE lncRNAs among developmental stages.

Supplementary Figure S4: Quantitative RT-PCR analysis of six representative lncRNAs.

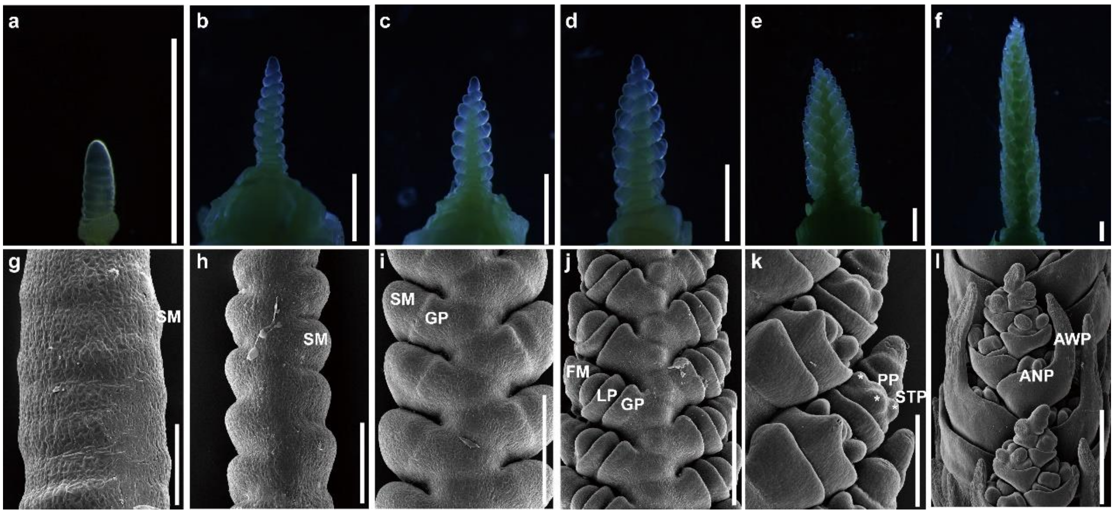

Supplementary Figure S1 Morphology of wheat spike at six developmental stages. **a-f** Morphology of spikes at the six developmental stages (S1-S6) under a stereomicroscope. **g-l** The scanning electron microscopy images of the spikes described in a-f. SM, spikelet meristem. GP, glume primordium. LP, lemma primordium. FM, floret meristem. STP, stamen primordium. PP, pistil primordium. ANP, anther primordium. AWP, awn primordium. Bars: a-f, 1mm; g, 0.1 mm; h, 0.2 mm; i, 0.3 mm; j, 0.4 mm; k, 0.3 mm; l, 0.5 mm.

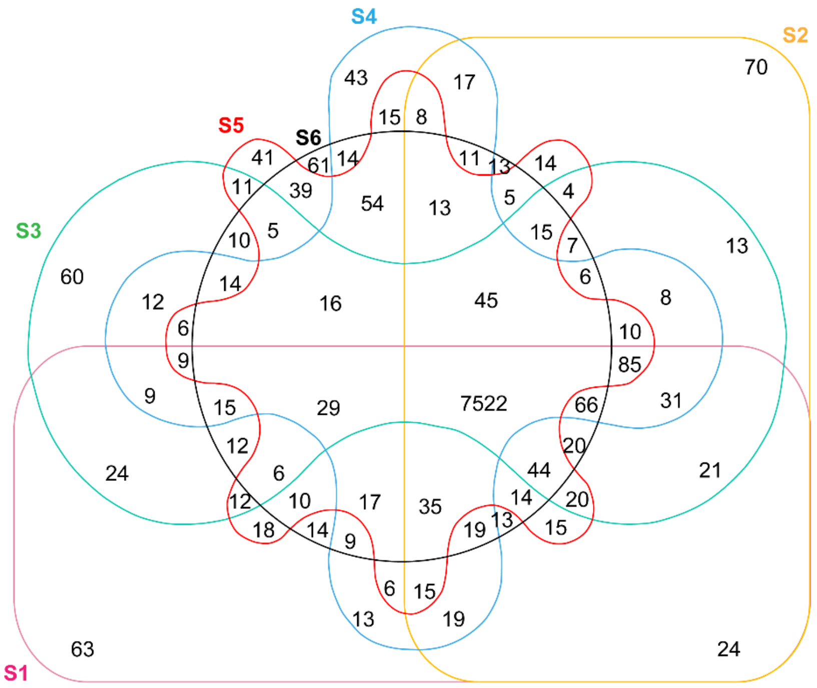

Supplementary Figure S2 Venn diagrams of the lncRNAs expressed in wheat spike. Six developmental stages (S1 to S6) are indicated by different colors.

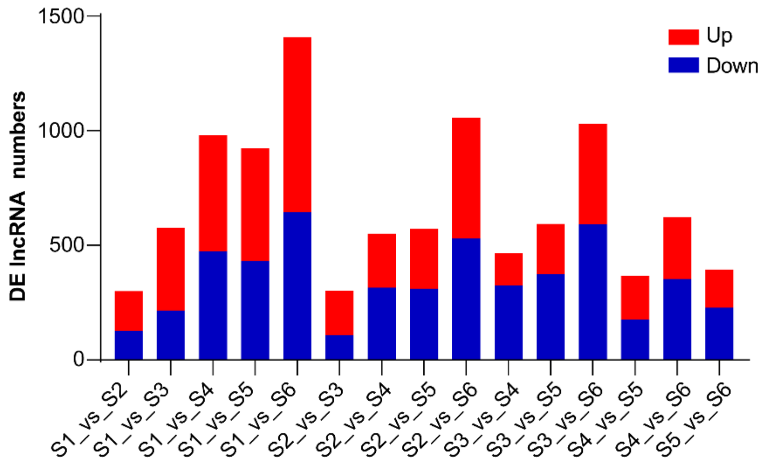

Supplementary Figure S3 Numbers of DE lncRNAs among developmental stages. The numbers of DE lncRNAs with increased or decreased expression between two compared stages are shown in red or blue, respectively. DE lncRNAs were filtered according to p-value < 0.05 and $\log_2$ (fold change) > 1 or < –1.

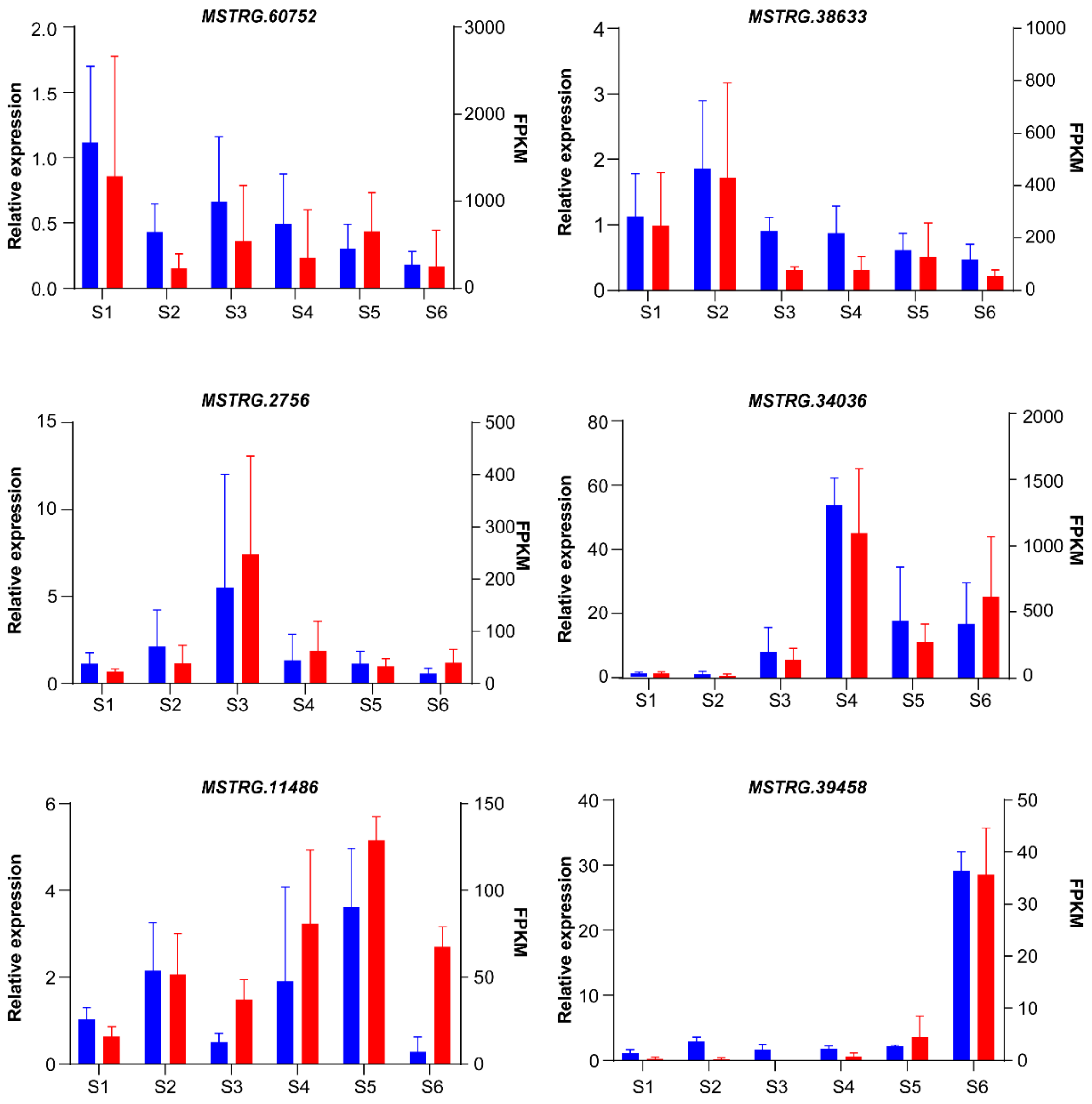

Supplementary Figure S4 Quantitative RT-PCR analysis of six representative lncRNAs. The expression levels of six representative lncRNAs are shown based on the data from qRT-PCR (blue) and RNAseq (red), respectively. The wheat *ACTIN* was used as an internal reference to normalize the qRT-PCR results. Data are from three biological replicates and error bars indicate SD.
